# Access and communication for deaf individuals in Australian primary care

**DOI:** 10.1111/hex.13336

**Published:** 2021-08-11

**Authors:** Phoebe H. Lee, Catherine Spooner, Mark F. Harris

**Affiliations:** ^1^ Faculty of Medicine and Health, Centre for Primary Health Care and Equity UNSW Sydney Sydney New South Wales Australia

**Keywords:** access, Australian Sign Language (Auslan), Deaf Community, equity, interpreters, primary health care

## Abstract

**Background and Aims:**

The Australian Deaf Community face barriers that impede their access to, and communication within, primary health care settings. This study aimed to identify barriers and facilitators to access and communication for deaf individuals and Auslan interpreters in Australian general practice settings.

**Methods:**

Semi‐structured interviews were conducted with eight Auslan interpreters and four deaf participants recruited from interpreter organisations and social media. Transcripts of interviews were coded inductively and deductively based on a model of access to health care.

**Results:**

Patient, provider and contextual factors were reported. Patient barriers included English and Auslan fluency levels within the Australian Deaf Community. GP clinics varied in the degree of accommodation to the needs of deaf people. There were barriers related to the communication methods used by health care providers and their use of interpreters. Visual aids and flexibility in terms of the GP clinics' appointment systems facilitated access. Contextual barriers included the shortage of Auslan interpreters and the complexity of the National Disability Insurance Scheme.

**Conclusion:**

The main barriers identified concerned the availability of interpreters, accommodation by health providers, cultural sensitivity and the adequacy of communication methods. Research is needed to explore the limitations of the National Disability Insurance Scheme and interventions to improve GPs' skills in communicating with Deaf individuals.

**Patient or Public Contribution:**

A researcher with a hearing impairment and experience in working with people with hearing impairments was consulted on study design and interview questions. Recruitment was assisted by Auslan interpreter agencies and a Deaf Community Facebook group.

## 1 INTRODUCTION

The Australian Deaf Community (ADC) consists of profoundly deaf or hard of hearing individuals who preferentially use Australian Sign Language (Auslan) to communicate.^1^ Johnston^1^ estimated that there were approximately 6500 Deaf signers in Australia in 2001, although this may be an underestimate as it did not account for deaf individuals who adopted use of Auslan later in their lives.^2^

There have been significant barriers to deaf individuals accessing primary health care and communicating with health care providers. These include the lack of text alternatives for phone‐based booking systems and the use of inadequate communication methods such as lip‐reading and written English.^3^, ^4^, ^5^, ^6^ The latter is problematic because written English is heavily dependent on the deaf individual's English literacy. Both international and Australian research has identified low English literacy levels within the Deaf Community and poor English literacy as the primary barriers to accessing preventive health information.^4^, ^5^, ^7^, ^8^, ^9^

Auslan interpreters provide a vehicle for communication between deaf individuals and their health care providers, but are employed infrequently in health care consultations.^9^, ^10^, ^11^ The Australian Disability Discrimination Act (1992) mandated equitable access to health care for deaf individuals, especially through the employment of Auslan interpreters where necessary. However, many health care providers lack knowledge of how to arrange Auslan interpreters and there is a shortage of interpreters across Australia.^8^, ^10^, ^12^ The shortage of Auslan interpreters is well documented, with the Australian Department of Social Services' 2004 survey of 491 deaf Auslan users identifying that 49% of deaf adults who had been to a doctor (GP or a specialist) had been unable to secure an interpreter in the preceding year.^10^ The NSW Deaf Society's interpreter service identified that it could not fill 79 requests for Auslan interpreting in serious medical, legal, mental health, social services and personal situations over a 1‐month period in 2014 under the National Disability Insurance Scheme (NDIS) scheme. The NSW Deaf Society attributed such workforce shortages to factors such as a high attrition rate of interpreters due to dissatisfaction with working conditions and the highly casualized nature of Auslan interpreting work. The average turnover period for an interpreter was equal to or less than that of the average time it took to train and accredit a new interpreter in 2015.^9^

There is a paucity of available research on the Deaf Community's access to health care in the Australian setting. This study thus aimed to explore the barriers and facilitators to both access and communication within the general practice setting experienced by both deaf individuals and Auslan interpreters in Australia.

## 2 METHODS

### 2.1 Sample and recruitment

#### 2.1.1 Auslan interpreters

Auslan interpreters who were fully accredited by the National Authority for Translations and Interpreters, had previous experience in interpreting in the primary health care and were older than 18 years of age were included in the study.

Interpreters were recruited via email from two different interpreting agencies: An Australia‐wide agency and an interpreting agency whose service was localized to a metropolitan area. All Auslan interpreters were hearing people (not hearing impaired). The recruitment of interpreters was stopped when thematic saturation was achieved.

#### 2.1.2 Deaf participants

The inclusion criteria for deaf individuals were that they had to be hearing‐impaired or profoundly deaf, used Auslan as their preferred mode of communication, culturally identified as a part of the Deaf Community and were older than 18 years of age. Deaf individuals who used sign languages other than Auslan (e.g., British Sign Language) and those who preferentially used spoken English as their preferred mode of communication were excluded.

*Consent process*: Interpreters and deaf people were sent the written participant information and consent forms via email and signed consent forms were sent back to the researcher before the interview. The information sheet invited participants to contact the researcher if they had any questions about the study and consent. None did so. All participants were asked if they had any questions before the interview. None did.

### 2.2 Data collection

The interview questions were developed on the basis of the relevant literature^3^, ^4^, ^6^, ^7^, ^8^, ^13^ and pilot‐tested with two participants (Appendix A). Similar questions were asked of the interpreters and the deaf participants. The questions investigated the barriers and facilitators of communication for the ADC in accessing GPs, personal experiences with the primary health care system and/or health providers and facilitators of communication with health care providers in the primary health care setting.

Qualitative interviews were conducted with Auslan interpreters and with deaf participants between June and August 2020. Semi‐structured interviews were conducted via telephone with Auslan interpreters. Interviews with deaf participants were conducted via online platforms with Auslan interpreters for translation. The researcher used spoken English for all interviews. The interview duration ranged from 30 to 60 min.

Ethics approval was granted by the UNSW's Human Research Ethics Committee (HC191007).

### 2.3 Data analysis

The audio recordings of interviews were transcribed, imported into Nvivo1218 and then coded inductively using the model of access to health care described by Levesque et al.^14^ Interpreter interviews were also coded deductively. This framework conceptualized access from the perspective of both patients and providers in five dimensions of provider‐side factors: (1) Approachability; (2) Acceptability; (3) Availability and Accommodation; (4) Affordability; and (5) Appropriateness and five corresponding patient‐side factors: (1) Ability to perceive; (2) Ability to seek; (3) Ability to reach; (4) Ability to pay; and (5) Ability to engage. The first three interviews were coded by all three authors together to check the coding framework and coding decisions. Subsequent interviews were coded by Phoebe H. Lee and 60% of the codes were checked by Mark F. Harris or Catherine Spooner. Disagreements in coding were discussed by the group of three authors.

## 3 RESULTS

### 3.1 Participants

All Auslan interpreters were fully certified with the National Accreditation Authority for Translators and Interpreters (Table 1). The deaf participants reported varying degrees of hearing loss. All deaf participants expressed a preference for Auslan as their primary method of communication (Table 2).

**Table 1:** Demographic information of Auslan interpreters

Interpreter	Sex	Age bracket	Interpreter experience (years)	State
1	F	40–60	20+	NSW
2	M	20–40	0–10	NSW
3	F	40–60	10–20	NSW
4	F	20–40	10–20	WA
5	F	20–40	10–20	Not provided
6	F	40–60	20+	WA
7	F	Not provided	Not provided	NSW
8	F	Not provided	Not provided	NSW

Abbreviations: F, female; M, male,

**Table 2:** Demographic information of deaf participants

Deaf participant	Sex	Age bracket	State
1	F	30–40	NSW
2	F	65+	QLD
3	M	30–40	VIC
4	M	20–30	NSW

Abbreviations: F, female; M, male.

#### 3.1.1 Approachability/ability to perceive

For deaf individuals who used Auslan as their primary language, English literacy was an important barrier to accessing and understanding health promotion material.

*If you go to the NSW Health department, you'll see heaps of information in languages other than Auslan. And same thing happened with COVID because the Federal Department of Homeland Security [sic], put out all this information in different community languages and not Auslan and I went up to them and said 'you gotta do it in Auslan, too'. And they're like, ‘Oh, no, we don't do that. Because we only do written languages, we can't do video’*. I7

Deaf individuals added that even if there was information available in Auslan, the information did not cater for the diversity of Auslan levels in the ADC.

*But sometimes, you know when you don't understand all. Even the news. It'll have the captions or they'll have the interpreter. He will still ask because it's not necessarily gearing to his level of communication. And sometimes the interpreter isn't clear with their signs, or we don't use that interpreter, there's too much in delay, and they're using the same signs, and the captions. You know when you compare the signs that they're using and the caption they're using I find it very difficult, but for a lot of Deaf people, it does go over their heads’*. P2

#### 3.1.2 Acceptability/appropriateness

Interpreters described instances where GPs used culturally inappropriate terms to refer to deaf individuals.

*Especially with a new doctor, like they hadn't seen their doctor before the deaf walked up with a sore foot or something. And the first thing the doctor would say is: Oh, have you thought about one of those bionic ears?* I7

*…I saw deaf people get really pissed off and it just completely ruins the relationship before it even starts. The doctor doesn't realize that they're saying something that's really. They don't do it on purpose. Or I've even had doctors use the term ‘deaf and dumb’. I've definitely had doctors say ‘hearing impaired’ which also pisses Deaf people [off] so but you can't get them across all that like you're not going to get them across every aspect. But I think a good interpreter will … say, I think the term you're looking for is deaf, would you like me to interpret that as deaf as to not cause offence or something like that?* I7

#### 3.1.3 Availability and accommodation

Both interpreters and deaf participants identified phone‐based appointment systems as a barrier to access for deaf individuals, with deaf participants stating that the National Relay Service (NRS) was inadequate to ensure prompt access.

*For me, the National Relay Service that I use to book would take quite some time. It's an exaggerated amount of time, which potentially then misses out on appointments. As a hearing person, they can get an appointment straightaway. So that's a big barrier for deaf people in terms of prompt access*. P4

SMS‐ and email‐based appointment systems were identified by deaf participants and interpreters as facilitators of access by interpreters and deaf participants.

*So, I just use the [proprietary] system, or email. I try not to use the NRS as much as I can. The reason being is signing isn't sufficient, most of the time*. P2

Before the introduction of the NDIS, GP clinics were responsible for booking interpreters. However, under the NDIS, it is the deaf individuals' responsibility to book their own interpreters for private medical appointments, including GP consultations. However, interviewees reported that the introduction of NDIS exacerbated the pre‐existing shortage of Auslan interpreters, an issue compounded by a lack of training programmes across a number of Australian states, as well as the absence of a specialized training pathway for Auslan interpreters to work in a medical setting.

*No, the booking system itself is relatively adequate. It's just literally a fact that there aren't enough of us. So, I would, on average before COVID happened, I'd say, I would say ‘no’ to about 90% of work and still be working full time*. I3

Even if interpreters were booked for an appointment, the waiting times at GP clinics meant that the interpreter would often have to leave before the appointment began.

*But now the waiting room is my biggest issue, because there's such delays at the GP. So when I finally get an interpreter, they often have to go because the wait time so long so it's a no win situation*. P1

Video remote interpreting (VRI) was identified by interpreters as a potential tool to overcome barriers concerning interpreter availability by interpreters by reducing ‘downtime’ getting to and waiting for interpreted appointments.

*So the interpreter drives all the way there, tries to find parking, navigates traffic, gets to the job, it's 10 min and then they're sitting in their car for, I don't know, an hour before the next job because they book an hour, my minimum is an hour and a half so we can't overlap that. So, so a lot of there was downtime in between jobs but now with VRI we're at home. And we can take up to 10 calls a day, which is, which is what's happening now that COVID has sort of subsided a bit*. I6

However, some deaf individuals reported that VRI was not always used to its full potential, resulting in an inability to see nonverbal communication.

*I can't see the doctors face when he's using his, when she's using her facial expressions. I'm only looking at the interpreter. Some deaf people don't mind, but most of my friends do, they'd like to be able to see that the interpreter with the, with the doctor side by side. And so that they could see how bad how serious, or how non‐bad or non‐serious it is because you can't see the doctor…. Others will use, and don't mind. But for me, I don't like it at all*. P2

#### 3.1.4 Affordability/ability to pay

NDIS was limited to deaf individuals under the age of 65. For individuals over the age of 65, interpreters can be booked through the National Australian Booking Service (NABS). One deaf participant expressed concern because this was limited to medical care and not available for aged care. Although NDIS has granted deaf individuals more autonomy in booking interpreters for the medical setting, several limiting factors were raised by interpreters and deaf participants. One such factor was the NDIS requirement that individuals estimate the funding that will be required for interpreters for medical appointments over the next year.

*They need to estimate how much funding they'll need for interpreters, or at least they'll need to give some indication. And it's practically impossible to give that*. I2

Another issue was that some clinics required double appointments to be booked when a patient was accompanied by an interpreter to allow extra time for communication. The patient was expected to pay the additional fee.

*I also have heard of some practices that recognize that it's good practice to book a longer appointment when interpreters are involved, but often I've heard of some of them wanting to pass the cost of that on to the deaf person so whereas they might, you know, the practice might bulk bill for standard length appointment. If I have to book a long appointment, they won't bulk bill that. They will charge a gap*. I8

#### 3.1.5 Appropriateness/ability to engage

A lack of cultural awareness of providers was reported to be a substantial barrier in communicating in the primary health care setting. Deaf participants reported that the most frequently used methods of communication in the absence of interpreters—lip‐reading and hand‐written notes—were inadequate.

*And some doctors I've seen out of pure luck I think will communicate with me using pen and paper. But their handwriting is not very easy to understand. So it's almost like in complete italics and I can't understand it or barely understand the sentence*. P1

Both interpreters and deaf participants identified barriers even in the presence of interpreters. Lack of provider knowledge about how to use Auslan interpreters affected the quality of communication, rapport between the provider and the patient and the effective use of time during the consultation. Limited time compounded this problem.

*I'm not finished explaining before the doctor sort of is giving more information, or the client hasn't finished signing to me, before you know, pushing in and asking more so a little bit more time and patience so always good for nice smooth interpreting appointment*. I5

Visual aids were discussed as an important facilitator of communication, especially because Auslan is a visual language.

*They like it when a doctor is able to use pictures to articulate what's going on. So if there's a word, they bring it up on the screen, so that you can then put two and two together*. P4

Deaf Awareness Training was frequently suggested as an intervention by both interpreters and deaf individuals to facilitate better communication. Interpreters discussed the potential of their roles as cultural navigators and/or educators.

In the absence of interpreters, deaf participants reported that family members would often interpret for them. However, they also reported that they expressed that this compromised their autonomy and privacy.

*My daughter, she can hear. She's now 11 years old. And she comes with me a lot of the time because I don't leave her at home on her own. And the doctor will go, ‘Oh, great. She's here’, and they treat her like an interpreter. But my daughter doesn't understand medical words. She's 11 years old. She doesn't understand the implications of what certain things mean. The name of medications are often long and complicated, and she doesn't understand that. So it's not something she feels comfortable doing either*. P1

## 4 DISCUSSION

This study aimed to explore the barriers faced by the ADC in accessing and communicating within the primary health care setting and found many such barriers.

There are few studies currently that discuss the perspective of and the challenges faced by Auslan interpreters. The strength of this study is that by exploring such challenges, it provides a foundation for addressing the inadequacy of both the quantity of Auslan interpreters in the health care setting in Australia and the quality of their training. Furthermore, this study was able to reveal a discrepancy between the deaf patients' needs and the interpreters' perceptions, elucidated by patients who forgo interpreter use, which is only possible with both the deaf participants' and the interpreters' involvement with the study.

The barriers were analysed using the Levesque access framework and are discussed below.

### 4.1 Approachability

Deaf participants and interpreters reported significant barriers to accessing the information on health services. Consistent with the literature, participants reported a diversity of English literacy levels and expertize in Auslan in the ADC.^15^, ^16^ Deaf individuals with low levels of Auslan skills, in the absence of sufficient English literacy required to understand health information, found health information that had been translated into Auslan difficult to understand. The variation in Auslan skills compounds the lack of health information available to the ADC, since most health information is currently unavailable in Auslan.^13^

### 4.2 Availability and accommodation

Participants reported barriers to using traditional phone‐based appointment systems, preferring text‐based appointment systems.^3^ This included not only arranging appointments but also follow‐up confirmation or rescheduling. Although the NRS was available as a speech‐to‐text converter for appointments arranged by phone, deaf participants reported that the service was time‐consuming, preventing timely access to often urgent care. A facilitator of access was the receptionist's familiarity with interpreters as well as the understanding that interpreters could only be present for a limited time, thus ensuring that the patients were able to be seen during the time that the interpreter was booked.

There were significant problems with the booking of Auslan interpreters. At the time this study was conducted, NDIS had superseded NABS for funding all medical interpreting services for deaf individuals under the age of 65. This shifted the responsibility of booking interpreters for GP consultations from the clinics to the deaf individuals themselves. Some interpreters identified this as a positive change, with patients being able to become more autonomous and empowered, as well as ensuring that the interpreters were requested for a future appointment. However, deaf participants are required to predict the amount of funding that they will require over the course of a year for medical interpreting. This can be impossible to predict, given that many health appointments are in response to new/unexpected health matters.

Although NDIS contributed to income stability for interpreters, it has also increased the number of nonmedical appointments for Auslan interpreters, reducing their availability for medical consultation. This issue has been compounded by the high attrition rate of Auslan interpreters due to poor working conditions, a highly casualized workforce and the restricted availability of training for Auslan interpreters.^10^ Strategies to address the shortage of Auslan interpreters include promoting Auslan interpreting as a genuine career pathway in secondary education, providing training for specialisation in interpretation in health care settings and retention of the current workforce through professional development, competitive remuneration and career flexibility.^17^

VRI has been suggested as a potential tool to help overcome interpreter shortage, especially in rural and remote areas.^18^ Interpreters in our study were consistently positive about the use of VRI, in particular noting that it could save travel time. Deaf participants, on the other hand, expressed mixed views. Some reported that doctors were sometimes unable to show themselves via video, so nonverbal communication could not be observed. This affected the ability to develop rapport with the doctor. Furthermore, the widespread adoption of VRI requires access to appropriate technology, the Internet and the skills to use it. Participants from both groups reported that the coronavirus disease 2019 (COVID‐19) pandemic had prompted a significant number of people to access and develop skills to use such technology, resulting in an increase in people's acceptance and skills in using VRI.

### 4.3 Appropriateness and ability to engage

Both interpreters and deaf participants discussed the inadequacy of communication methods commonly used by health care providers, namely, lip‐reading and written English, in the absence of interpreters. The insufficiency of lip‐reading and written English as communication methods with deaf individuals has been well documented in the literature.^4^, ^5^, ^8^, ^13^ Previous studies suggested that both patients and providers overestimate the efficacy of these communication skills, when deaf people typically understand less than 30% of what an individual says through lip‐reading, and instead gain understanding from contextual clues. Cultural sensitivity and visual aids were highlighted as two of the most prominent factors in ensuring that first, the deaf participant could communicate fully to the provider, and second, the provider can ensure the understanding of crucial information. Family members were often inappropriately asked to undertake the roles of interpreters. However, relatives not only risk inaccurate interpretation or withholding of information but their use also impinges upon the privacy and autonomy of the patient.^8^, ^9^

Even when interpreters were available, the use of fully certified Auslan interpreters was insufficient to ensure complete understanding and a satisfying doctor–patient interaction. A significant proportion of health concepts do not have an Auslan sign.^19^ Therefore, Auslan interpreters have often been required to ‘unpack’ the meaning of medical terms to the deaf patients. Furthermore, specific training for interpreting in the medical setting is not included in Auslan; interpreters currently do not train specifically for the medical setting, which can mean that more time is required to fully interpret the meaning. Compounding the problem, some clinics would require double bookings for patients accompanied by interpreters, with the patient incurring an additional fee.

Deaf Awareness Training for helth care providers was highlighted as the key interventional strategy by deaf participants and interpreters. Deaf Awareness Training in Australia is conducted by local Deaf Societies, usually by a deaf person, and seeks to raise cultural awareness and the communication needs of the ADC.^2^

### 4.4 Implications and further research

This study described the barriers that the ADC has faced in their access to and communication within the primary health care system. However, further research is required to ascertain the facilitators that can mitigate such barriers, especially regarding the national, systematic shortage of interpreters.

Furthermore, this study shows a discrepancy even among the ADC in their experiences and perceptions of health care. There is considerable variation between deaf individuals for factors such as level of hearing impairment, age of sign language acquisition and whether they were raised in a hearing or a Deaf environment. By stratifying data according to these factors, new themes may emerge.

Future research could also extend beyond primary health care and explore the needs of the ADC in the broader health care system, including tertiary and emergency health care.

### 4.5 Limitations of the project

Deaf participants were all recruited online, and only online interviews were possible because of restrictions due to the COVID‐19 pandemic at the time. This may limit the generalizability of our findings, especially for individuals without access to or skills in using online technology. The small number of deaf participants recruited meant that we were unable to reach data saturation, so further research with deaf participants would be valuable.

It is possible that there was a negative bias in the study results. Deaf individuals were very vocal about their negative experiences with their access to health care access. The individuals were recruited from an ADC social media page, which individuals have been observed to utilize to discuss negative experiences that they have had as hearing‐impaired persons. Interviews were conducted in an inductive manner. As more themes surrounding negative experiences of deaf individuals had emerged, less questions were subsequently focused on exploring positive experiences regarding the health care system.

## 5 CONCLUSION

It is known that the ADC has faced significant barriers to accessing primary health care due to the lack of availability and use of Auslan interpreters, insufficient cultural knowledge, health care providers' attitudes regarding the ADC and systemic barriers. This study contributed knowledge about these barriers by identifying, for example, appointment systems that did not accommodate the needs of deaf people, inadequate availability of interpreters, culturally inappropriate GP practices and ineffective GP communication methods. The study described how these barriers can be addressed through tools such as visual aids and text‐based clinic appointment systems. Strategies to address systemic access barriers were identified. These included increased resources and training for interpreters to work competently in the health sector and changes to how the NDIS system funds individuals for Auslan interpretation in primary health care settings.

More research is needed to inform ways to increase the interpreter workforce and to improve the skills of primary care providers to use them. There is also a need for research to inform how to improve NDIS‐funded access to interpreters for the ADC.

## CONFLICT OF INTERESTS

The authors declare that there are no conflict of interests.

## AUTHOR CONTRIBUTIONS

Phoebe H. Lee conducted the entire data collection and wrote the first draft of the article under the supervision of Catherine Spooner and Mark F. Harris. Phoebe H. Lee conducted all interviews as part of a research elective for medical training. Phoebe H. Lee was trained and supervised by Mark F. Harris and Catherine Spooner. All three authors made substantial contributions to the conception and design, analysis and interpretation of data; were involved in revising the article critically for important intellectual content; have given final approval of the version to be published and have participated sufficiently in the work to take public responsibility for appropriate portions of the content; and have agreed to be accountable for all aspects of the work in ensuring that questions related to the accuracy or integrity of any part of the work are appropriately investigated and resolved.

## Data Availability

The data that support the findings of this study are available on request from the corresponding author if approval is obtained from the UNSW Human Research Ethics Committee. The data are not publicly available due to privacy or ethical restrictions.

## APPENDIX A AUSTRALIAN SIGN LANGUAGE AND ACCESS TO PRIMARY HEALTH CARE

Interview questions for deaf participants

1. Do you think that deaf people experience barriers in accessing/booking general practice appointments, especially when compared with the hearing population? If so, what do you see as the main barriers?
2. Tell us about your own experiences in accessing and seeing a GP.
3. Is there anything that helps or makes it easier for deaf people in accessing a GP?
4. Tell us about your experiences when communicating with a GP.
5. Is there anything that helps or makes it easier for deaf people to communicate with a GP?
6. What have you found helpful in communicating more easily?
7. What do you see as the main barriers for deaf people communicating with general practice staff?
8. What about your own experiences in communicating with general practice staff or GPs?
9. How do you think the health care system could better respond to the needs of the Deaf community?
10. Is there anything more you would like to say or ask?

Australian sign language and access to primary health care

Interview questions for interpreters

1. What do you see as the main barriers in accessing general practice for deaf people?
2. Is there anything that helps or makes it easier for deaf people to see a GP?
3. What do you see as the main barriers for deaf people in communicating with general practice staff?
4. What have you found helpful in making communication easier between the patient and the GP?
5. (For interpreters) As an interpreter, how effective is the current National Disability Insurance Scheme/booking system for the GP setting?
6. How do you think the health care system could better respond to the needs of the Deaf community?
7. Is there anything more you would like to say or ask?
